# Case Report: Mitochondrial Encephalomyopathy Presents as Epilepsy, Ataxia, and Dystonia With a Rare Mutation in *MT-TW*

**DOI:** 10.3389/fneur.2021.679302

**Published:** 2021-07-01

**Authors:** Shuang Wang, Jing Miao, Jiachun Feng

**Affiliations:** Department of Neurology, The First Hospital of Jilin University, Changchun, China

**Keywords:** mitochondrial encephalomyopathy, *MT-TW*, epilepsy, ataxia, dystonia

## Abstract

Mitochondrial diseases are a group of common inherited disorders caused by mutations in nuclear DNA or mitochondrial DNA (mtDNA); the clinical phenotype of diseases caused by mutant mtDNA is challenging owing to heteroplasmy of mtDNA and may delay diagnosis and treatment. Herein, we report the case of an adult male who slowly developed epilepsy, ataxia, dystonia, impaired cognition, and hearing impairment over 14 years in the absence of clinical myopathy. His lactate level was normal. Brain computed tomography showed calcifications of the bilateral basal ganglia, thalamus, and cerebellar dentate nuclei. Magnetic resonance imaging revealed multiple lesions in the bilateral internal capsule and periventricular areas, which were hypointense on T1-weighted images and hyperintense on T2-weighted images. The first blood genetic test result was negative. Two years later, a muscle biopsy was performed. Succinate dehydrogenase (SDH) staining showed several ragged blue fibers and atypical strongly SDH-reactive vessels. Cytochrome C oxidase (COX) staining revealed abundant COX-deficient fibers. mtDNA testing of blood and muscle revealed a rare m.5549G>A mutation in the *MT-TW* gene. It was heteroplasmic, with 5.4% mutant mtDNA in the blood and 61.5% in the muscle. The patient was diagnosed with mitochondrial encephalomyopathy and treated with levetiracetam instead of valproate to reduce possible mitochondrial toxicity. After receiving anti-epileptic drugs and mitochondrial supplements, the patient remained clinically stable. For mitochondrial disease, when mutant mtDNA is not detected in blood, muscle biopsy should be performed in routine analysis, and it should be genetically tested, even if there are no manifestations of myopathy.

## Introduction

Mitochondrial diseases are caused by mutations in nuclear DNA (nDNA) or mitochondrial DNA (mtDNA) and are characterized by dysfunction of oxidative phosphorylation (OXPHOS) and energy metabolism. Autosomal, X-linked, and maternal inheritance are all patterns of inheritance seen with mitochondrial diseases. It has been estimated that the prevalence of childhood-onset (<16 years) mitochondrial disease is 5–15 cases per 100,000 people, and in adults, the prevalence of causative mtDNA and nDNA mutations is estimated at 2.9 and 9.6 per 100,000 individuals, respectively (1, 2). The onset of mitochondrial diseases has a bimodal distribution, with one peak before 3 years of age and the other at the end of adolescence to 40 years of age (2).

Mitochondrial diseases can involve any organ but especially affect organs that depend on aerobic metabolism, and these present with a range of symptoms. These diseases are clinically heterogeneous and characterized by epilepsy, progressive muscle weakness, dementia, ataxia, peripheral neuropathy, optic atrophy, hearing loss, stroke-like episodes, and diabetes mellitus. Some of the clinical features can be grouped into specific syndromes, such as Leigh syndrome, Kearns–Sayre syndrome, myoclonic epilepsy myopathy sensory ataxia, mitochondrial encephalomyopathy with lactic acidosis and stroke-like episodes syndrome (MELAS), and myoclonic epilepsy with ragged red fibers (RRFs) syndrome. Common neuroimaging findings include white- or gray-matter lesions, atrophy, optic atrophy, stroke-like lesions, calcifications, and ischemic stroke (3). The diagnosis is complicated by variations in clinical phenotype and genotype. Currently, considerable advances in genetic testing technologies have been made, and most mutations in mtDNA can be detected in blood or urinary sediment. However, because mutant mtDNA heteroplasmy can vary across tissues (2), histochemical and biochemical analyses of tissue biopsies are essential for patients who have not been diagnosed by genetic testing of blood or urine samples.

Here, we report a case of adolescent-onset and slowly progressive mitochondrial encephalomyopathy in the absence of clinical myopathy caused by a rare m.5549G>A mutation in the *MT-TW* gene, which was diagnosed late due to the first negative blood mtDNA test finding that subsequently led to treatment delay.

## Case Report

**Figure 1:**
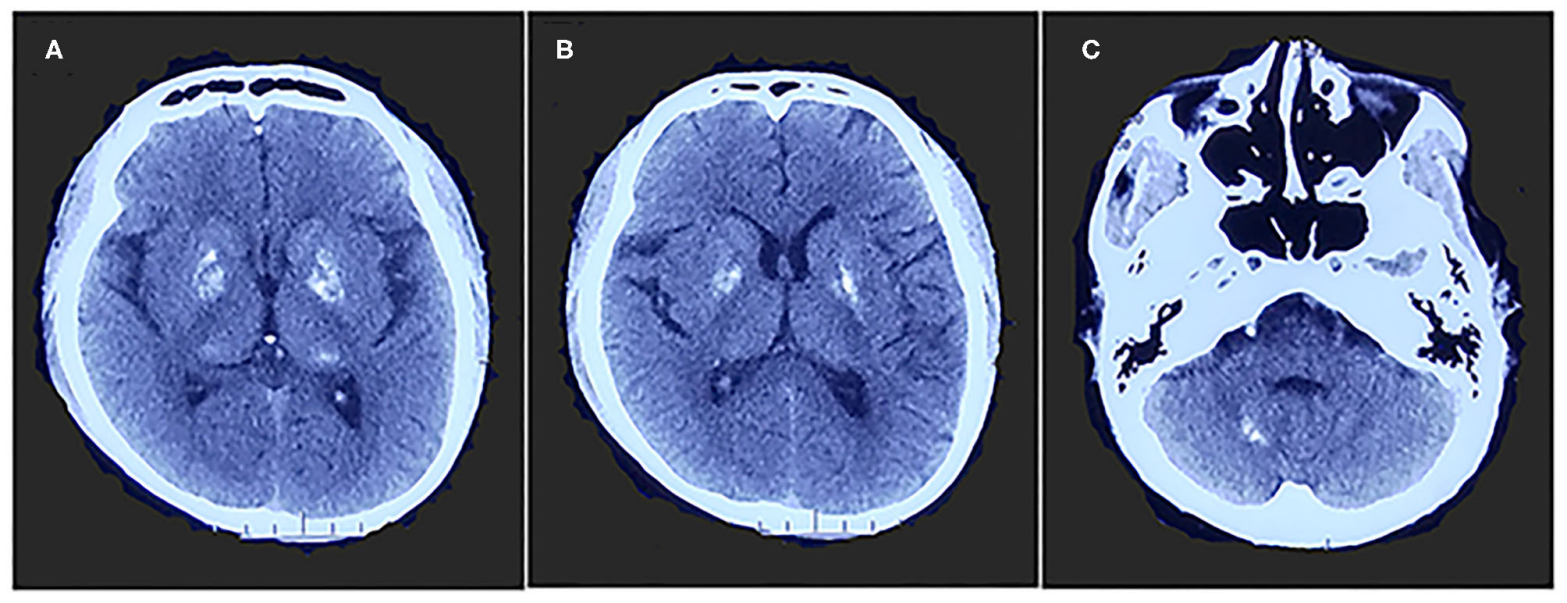
Brain computed tomography shows calcifications of bilateral basal ganglia, thalamus **(A,B)**, and cerebellar dentate nuclei **(C)**.

**Figure 2:**
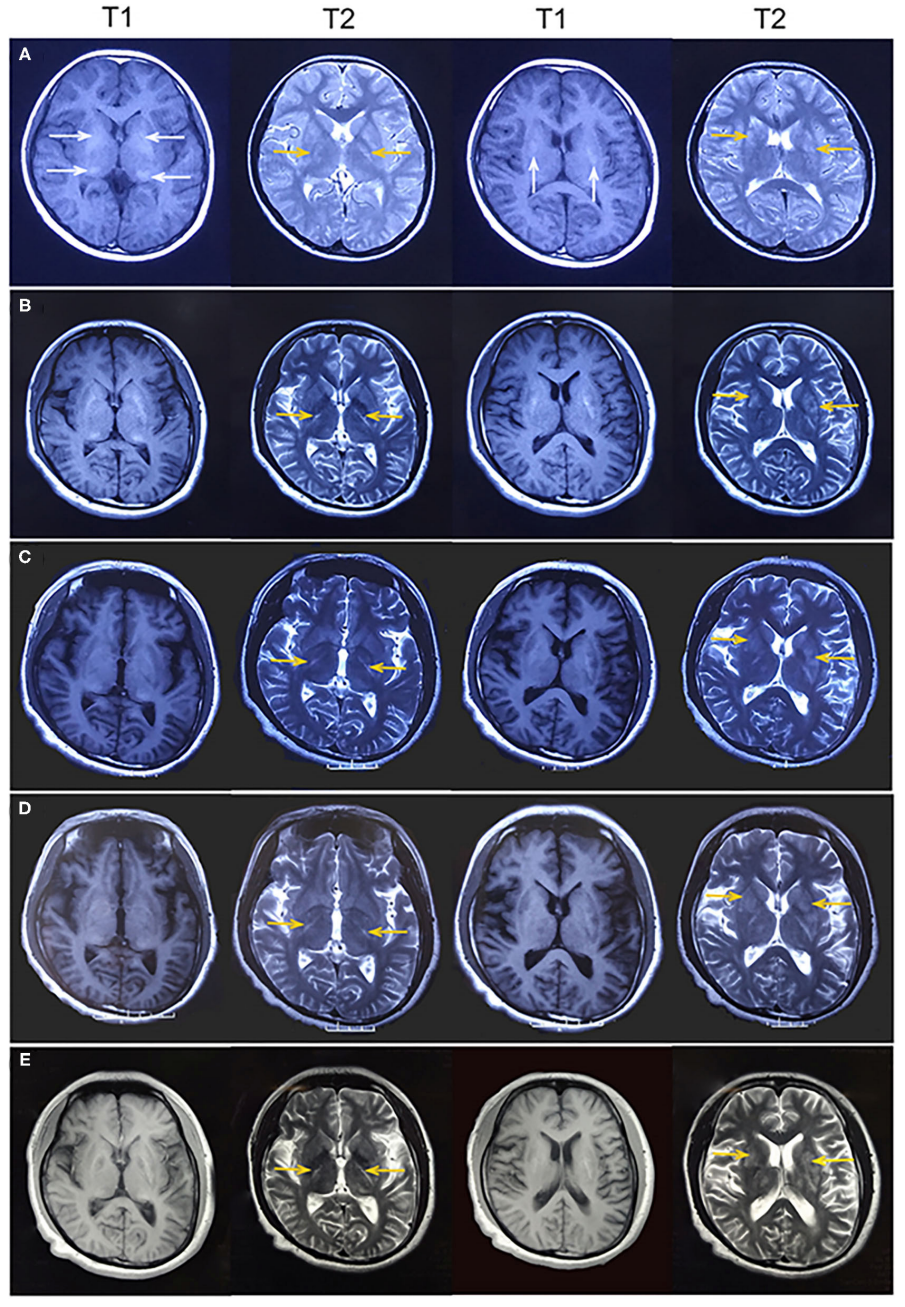
**(A)** At age 12 years, magnetic resonance imaging (MRI) revealed hyperintense on T1-weighted images and hypointense or hyperintense on T2-weighted images in the bilateral basal ganglia and thalamus (white arrowhead), which were coincident with areas showing calcifications on computed tomography. Some lesions were slightly hypointense or isointense on T1-weighted images, with T2 hyperintense in the bilateral internal capsule and periventricular areas (yellow arrowhead). **(B)** At age 19 years, MRI showed mild brain atrophy, but the lesions of bilateral internal capsule and periventricular areas (yellow arrowhead) did not change significantly compared to those in the previous MRI. **(C–E)** At age 22, 24, and 26 years, MRI showed no change in the lesions of bilateral internal capsule and periventricular areas (yellow arrowhead). Brain atrophy showed no obvious change.

A 26-year-old right-handed man presented with epilepsy, progressive worsening of gait imbalance, and involuntary movement for approximately 14 years. He had non-consanguineous parents and no relevant family history of neurological disease. When he was 12 years old, he experienced a tonic–clonic seizure. At that time, electroencephalography (EEG) showed extensive slow waves, especially in the posterior part of the brain. Brain computed tomography (CT) showed calcifications of the bilateral basal ganglia, thalamus, and cerebellar dentate nuclei (Figure 1). Magnetic resonance imaging (MRI) revealed hyperintense on T1-weighted images and hypointense or hyperintense on T2-weighted images in the bilateral basal ganglia, thalamus, and cerebellar dentate nuclei, which were coincident with areas showing calcifications in the CT images. The other lesions were slightly hypointense or isointense on T1-weighted images, with T2 hyperintense in the bilateral internal capsule and periventricular areas (Figure 2A). There were four episodes in the following 6 years. At the age of 18 years, he developed frequent absence seizures, and EEG showed generalized, symmetric, 3- to 3.5-Hz spike-and-wave discharge. The frequency of attacks was variable, but he did not receive any treatment. One year later, he developed gait instability and walked with a wide-based gait. MRI showed mild brain atrophy, but the lesions did not change significantly compared to those in the previous MRI (Figure 2B). His ataxia gradually worsened, and his parents found that he had developed impaired cognition. At the age of 22 years, his EEG showed generalized, low-to-medium amplitude, 3- to 4-Hz spike-and-wave discharge, and lesions in the MRI were unchanged (Figure 2C). He underwent blood genetic testing for the ataxia panel, which was negative, and he was treated with 500 mg of valproate twice daily. His clinical seizures were effectively controlled, but the ataxia and cognitive function did not improve.

**Figure 3:**
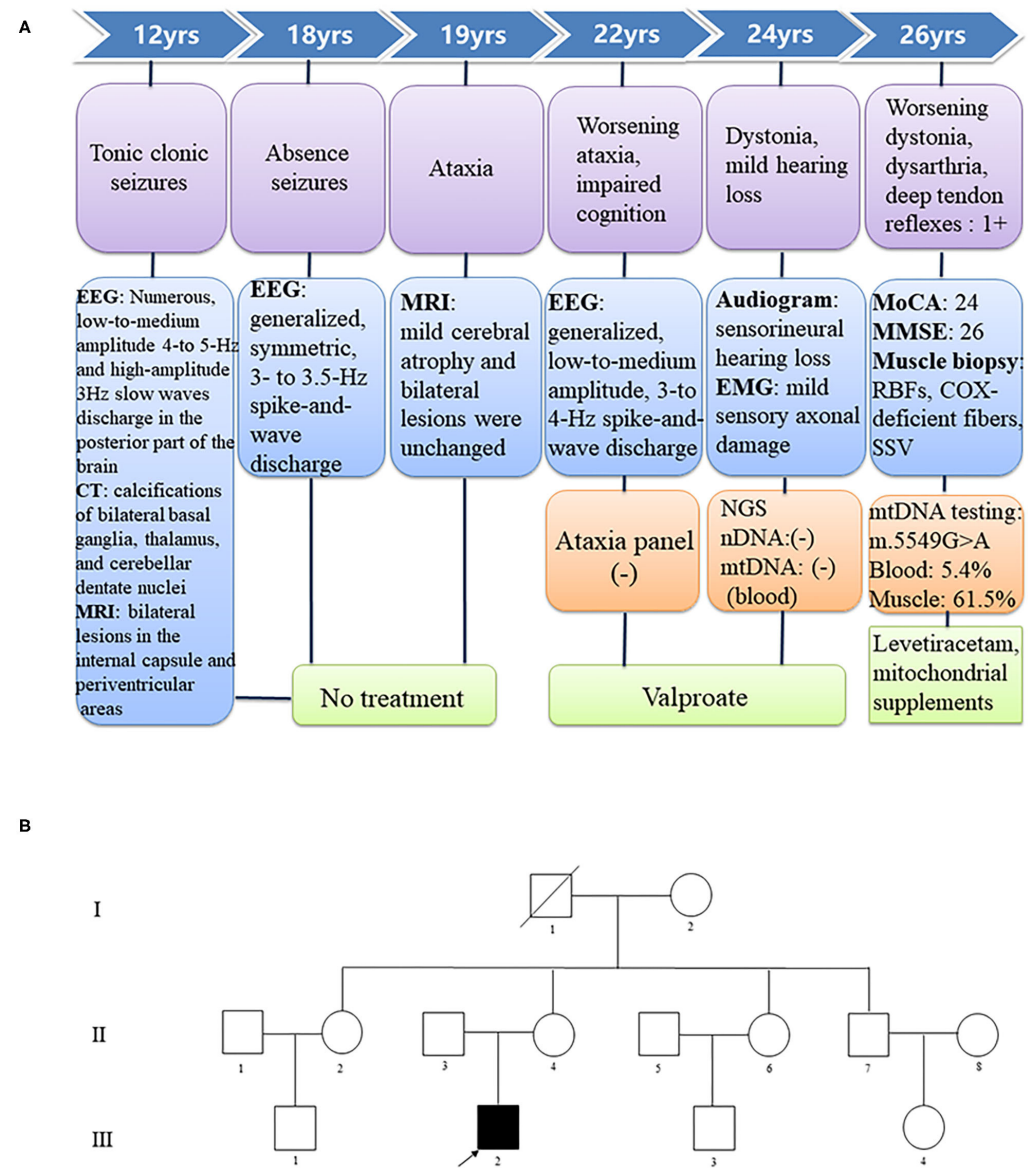
**(A)** Chronology of the major clinical features. EEG, electroencephalography; CT, computed tomography; MRI, magnetic resonance imaging; EMG, electromyography; MoCA, Montreal Cognitive Assessment; MMSE, Mini-Mental State Examination; RBFs, ragged blue fibers; SSV, strongly succinate dehydrogenase-reactive vessel; COX, cytochrome C oxidase; NGS, next-generation sequencing; nDNA, nuclear DNA; mtDNA, mitochondrial DNA. **(B)** Pedigree of the family showing clinically and genetically affected members (black squares).

At age 24 years, he presented with involuntary movements of his hands and mouth. The involuntary movement of the left hand was more severe than the right hand and manifested as a backward swinging motion, which was more obvious when he was holding objects. In addition, the patient suffered from mild hearing loss. Laboratory findings for blood electrolytes, parathyroid hormone, copper, ceruloplasmin, and lactate levels were normal. An audiogram showed bilateral sensorineural hearing loss. Neurophysiological studies indicated mild sensory axonal damage. Echocardiography and electrocardiography findings were normal. MRI showed no change in the lesions (**Figure 2D**). Next-generation sequencing (NGS) indicated no definite pathogenic mutations in nDNA or mtDNA of blood. In the following 2 years, the patient's involuntary movement gradually worsened. At age 26 years, he visited our clinic with the abovementioned symptom (Figure 3). The patient denied weakness and bladder or bowel dysfunction. Neurological examination revealed dysarthria and no nystagmus. His limb strength was normal. Deep tendon reflexes were 1+. Bilateral pyramidal signs were positive, and no sensory disturbances were detected. A mental examination revealed an orientation obstacle. The Montreal Cognitive Assessment score was 24 and the Mini-Mental State Examination score was 26. Routine blood tests and laboratory examinations for creatine kinase, phytanic acid, amino acids, acylcarnitine, and lactate levels revealed normal findings. Urine test results for organic acids were also normal. Serologic tests for syphilis and human immunodeficiency virus showed negative results. There were no acanthocytes in the peripheral blood smear. The lesions on MRI were the same as before (Figure 2E). Magnetic resonance spectroscopy (MRS) revealed no lactate peaks.

**Figure 4:**
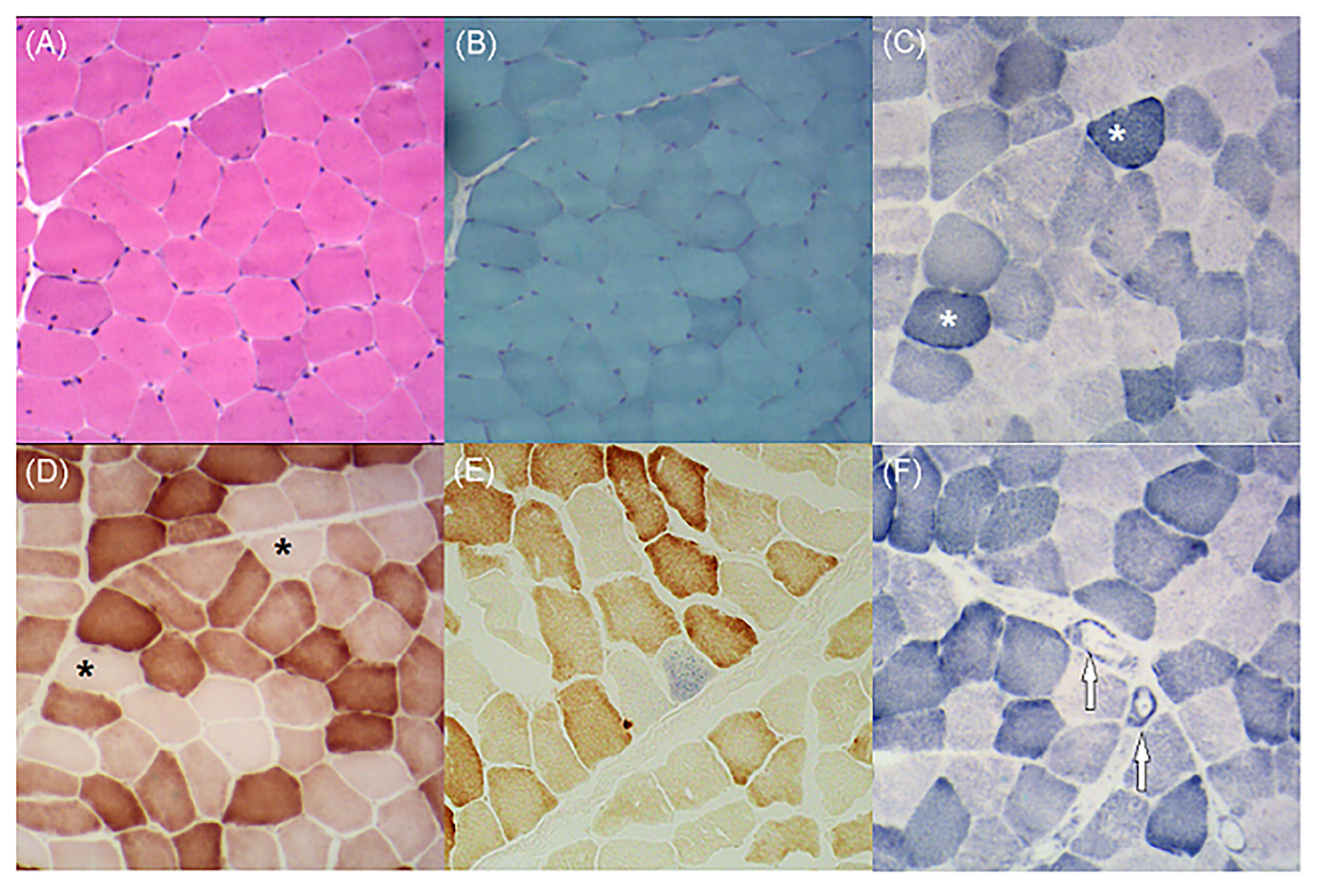
**(A)** Hematoxylin and eosin staining demonstrates no obvious abnormalities. **(B)** Modified Gomori trichrome staining shows no ragged red fibers. **(C)** Succinate dehydrogenase (SDH) staining shows several ragged blue fibers (asterisks). **(D)** Cytochrome C oxidase (COX) staining shows plenty of COX-deficient fibers (asterisks). **(E)** COX-SDH staining shows occasional COX-negative and SDH-positive fibers in blue. **(F)** SDH staining shows atypical strongly SDH-reactive vessels (arrows).

As the patient had adolescent onset of symptoms and slowly developed epilepsy, ataxia, dystonia, impaired cognition, and hearing impairment along with the characteristic findings of symmetrical basal ganglia calcifications, leukoencephalopathy, and cerebral atrophy, mitochondrial disorder was suspected despite negative mtDNA blood test findings. Given that mutant mtDNA heteroplasmy can vary across tissues and defects in mtDNA typically present in post-mitotic tissues (2), we performed a biceps brachii biopsy after obtaining written consent from the patient. Hematoxylin and eosin staining showed no obvious abnormalities. Typical RRFs were not observed in modified Gomori trichrome staining. Succinate dehydrogenase (SDH) staining revealed ragged blue fibers (RBFs) and atypical strongly SDH-reactive vessels (SSVs). Cytochrome C oxidase (COX) staining revealed abundant COX-deficient fibers. COX-SDH staining showed only occasional COX-negative and SDH-positive fibers in blue (Figure 4). mtDNA testing of blood and muscle biopsy sample was performed, which detected a known m.5549G>A mutation in the *MT-TW* gene (4). It was heteroplasmic, with 5.4% mutant mtDNA in the blood and 61.5% in the muscle. The patient was diagnosed with mitochondrial encephalomyopathy. I_2_, II_4_, and II_7_ agreed to carry out genetic testing of blood, but no mutations were detected in their blood.

The patient was previously treated with valproate. Considering its mitochondrial toxicity, we replaced valproate with levetiracetam. Mitochondrial supplements such as coenzyme Q10 (600 mg/day) and vitamins B2 (100 mg/day), B3 (250 mg/day), C (600 mg/day), and E (300 mg/day) were also administered. The patient was anxious before, but after the diagnosis of mitochondrial encephalomyopathy, he actively cooperated with the treatment and performed rehabilitation exercises. At the 1-year follow-up, he showed improvement in dystonia and ataxia, which remained clinically stable. As the symptoms stabilized, the increase in confidence of the patient was also conducive to treatment.

## Discussion

This case showed adolescent onset of symptoms and slow development of epilepsy, cerebellar ataxia, dystonia, impaired cognition, and hearing loss. He had characteristic imaging findings, including intracerebral calcification, white matter lesions, and brain atrophy. In muscle biopsy, RBFs, SSVs, and COX-deficient fibers were detected. In addition, a rare m.5549G>A heteroplasmic mutation in *MT-TW* was detected in the blood and muscle. All these manifestations were in line with the characteristics of mitochondrial diseases, and the patient was finally diagnosed with mitochondrial encephalomyopathy.

Our patient had abundant COX-deficient fibers in COX-stained samples. This is consistent with the characteristics of mutations in *MT-TW*. The *MT-TW* gene encodes the mitochondrial tRNA for tryptophan (tRNATrp). More than 10 mutations in *MT-TW* have been reported to date (5). Mutations in *MT-TW* may interfere with the structure and stability of tRNATrp, which can decrease the rate of mitochondrial protein synthesis and result in OXPHOS deficiency. Profound complex IV defects are a common feature of most tRNATrp mutations, which may be related to the higher percentage of tryptophan in the COI and COIII subunits (6, 7). Mutations in *MT-TW* can give rise to MELAS, neurogastrointestinal syndrome, Leigh syndrome, and mitochondrial myopathy (8–10).

The m.5549 G>A mutation in *MT-TW* is rarely found in mitochondrial diseases. In 1995, a patient reported to have the m.5549G>A mutation presented with progressive dementia, chorea, cerebellar ataxia, deafness, and peripheral neuropathy in the absence of clinical myopathy (4). That patient's symptoms began at the age of 40 years, developed rapidly and seriously, and led to death at the age of 53 years. His plasma lactate concentration increased after exercise, and CT showed severe atrophy of the whole brain with low-density lesions in the cerebral white matter. Muscle biopsy specimens showed COX-negative fibers, RRFs, and reduced complex I activity on polarography. Post-mortem examination showed heteroplasmic mutant mtDNA distributed in all tissues, ranging from 40% mutant mtDNA in blood to 93% in cardiac muscle. The proportions in the muscle and cerebral cortex were 92 and 87%, respectively. Compared with that patient, our patient differed in terms that he had adolescent onset, slow development of disease, and normal lactate levels. In his 14-year medical history, there was mild cerebral atrophy and symmetrical calcification. Bilateral lesions in the bilateral internal capsule and periventricular areas remained stable, providing additional imaging features for mitochondrial diseases. Muscle biopsy did not show RRFs, but RBFs, SSVs, and COX-deficient fibers were detected. In addition, the proportions of heteroplasmic mutations in the blood and muscle were 5.4 and 61.5%, respectively. The two cases have similarities in terms of clinical manifestations, but the age of onset, disease severity, and prognosis were obviously different. The difference between the two cases may be related to the proportion of mtDNA mutations in the affected tissue and the fact that the susceptibility of tissues to specific defects may change with the age of the patient (2). Both cases had a high percentage of mtDNA mutations in muscle in the absence of clinical myopathy and mainly presented with encephalopathy. These may be characteristics of the m.5549 G>A mutation in *MT-TW*. In our case, an interesting phenomenon is that the patient was clinically progressing but the brain MRI lesions seem to be stable. The brain MRI lesions existed 14 years ago, but he did not show ataxia or dystonia at that time. We speculate that with the development of the disease, the increase in the proportion of mutant mtDNA, the application of mitochondrial toxic drugs, and the long-term mental stress of the patient gradually revealed the patient's symptoms. In addition, MRS revealed no lactate peaks in our patient, because MRS is more sensitive to detect the lactate peak in the acute phase of mitochondrial disease. In patients with chronic MRI lesions, MRS may not detect the lactate peak (11).

The key features of our patient were cerebellar ataxia and dystonia. Their causes can be divided into acquired and genetic etiologies. In general, acquired causes can be ruled out based on laboratory tests or neuroimaging. Genetic testing is a valuable tool for determining the genetic etiology. When considering a certain disease, we can perform a relevant panel test. If the panel is negative or we are not sure about the diagnosis, NGS can be considered. For mitochondrial disease, the primary choice of tissue for genetic testing is blood, as harmful mitochondrial tRNA mutations may be actively eliminated in rapidly dividing cells (12). When mutant mtDNA is not detected in the blood, muscle biopsy should be performed in routine analysis, and these samples should be genetically tested, even if there are no manifestations of myopathy (13). Our patient had no mtDNA mutation detected in the blood at the first mtDNA testing, whereas a mutation was detected 2 years later, but the proportion of mutations was only 5.4%. An increase in the proportion of mtDNA mutations in tissues may be attributed to disease progression. Since his first blood test was negative and a tissue biopsy was not performed, the diagnosis and treatment of the disease was delayed.

Currently, treatments for mitochondrial diseases are intended to slow down progression and alleviate symptoms. No Food and Drug Administration-approved drugs are presently available for the treatment of mitochondrial diseases (14). In addition to dietary regulation and mitochondrial supplementation, reducing the use of drugs with mitochondrial toxicity can delay disease progression. Valproate, carbamazepine, phenytoin, and phenobarbital are anti-epileptic drugs with mitochondrial toxicity (15). Valproate can isolate coenzyme-A and cytochrome-aa3, inhibit key enzymes of β-oxidation, and cause damage to the inner mitochondrial membrane and secondary carnitine deficiency. In addition, it can result in complex I and complex IV dysfunction and decreased ATP production (16). In this case, the patient was treated with valproate for 4 years before diagnosis, and we replaced valproate with levetiracetam. For mitochondrial diseases, gene therapy is still a popular research topic, and stem cell-derived mitochondrial transplantation has been shown to play a key role in metabolic rescue, which provides promise for mitochondrial encephalomyopathy (17). To date, there are a total of 49 registered clinical trials of new experimental drugs for the treatment of mitochondrial diseases, and 10 are Phase III trials. However, as of 2019, only one has been completed and the results have not been reported (14), and the treatment of mitochondrial diseases still faces many challenges.

## Conclusions

This case demonstrates a rare mutation in *MT-TW* associated with epilepsy, ataxia, and dystonia in the absence of clinical myopathy, which was diagnosed late due to the first negative blood mtDNA test finding. For mitochondrial diseases, when blood mtDNA test result is negative, muscle biopsy should be performed in routine analysis, even if there are no manifestations of myopathy.

## Data Availability Statement

The original contributions presented in the study are included in the article/supplementary material, further inquiries can be directed to the corresponding authors.

## Ethics Statement

Written informed consent was obtained from the individual(s) for the publication of any potentially identifiable images or data included in this article.

## Author Contributions

JF and JM contributed to the conception and design of the manuscript. SW wrote the manuscript. All authors contributed to manuscript revision, read, and approved the submitted version.

## Conflict of Interest

The authors declare that the research was conducted in the absence of any commercial or financial relationships that could be construed as a potential conflict of interest.
